# Response of plants to water stress

**DOI:** 10.3389/fpls.2014.00086

**Published:** 2014-03-13

**Authors:** Yuriko Osakabe, Keishi Osakabe, Kazuo Shinozaki, Lam-Son P. Tran

**Affiliations:** ^1^Gene Discovery Research Group, RIKEN Center for Sustainable Resource ScienceTsukuba, Japan; ^2^Center for Collaboration among Agriculture, Industry and Commerce, The University of TokushimaTokushima, Japan; ^3^Signaling Pathway Research Unit, RIKEN Center for Sustainable Resource ScienceYokohoma, Japan

**Keywords:** abiotic stress, biomass, drought stress, photosynthesis, reactive oxygen species, stomatal closure

## Abstract

Water stress adversely impacts many aspects of the physiology of plants, especially photosynthetic capacity. If the stress is prolonged, plant growth, and productivity are severely diminished. Plants have evolved complex physiological and biochemical adaptations to adjust and adapt to a variety of environmental stresses. The molecular and physiological mechanisms associated with water-stress tolerance and water-use efficiency have been extensively studied. The systems that regulate plant adaptation to water stress through a sophisticated regulatory network are the subject of the current review. Molecular mechanisms that plants use to increase stress tolerance, maintain appropriate hormone homeostasis and responses and prevent excess light damage, are also discussed. An understanding of how these systems are regulated and ameliorate the impact of water stress on plant productivity will provide the information needed to improve plant stress tolerance using biotechnology, while maintaining the yield and quality of crops.

## INTRODUCTION

Plant growth and productivity are adversely affected by water stress. Therefore, the development of plants with increased survivability and growth during water stress is a major objective in the breeding crops. Water use efficiency (WUE), a parameter of crop quality and performance under water deficit is an important selection trait. In fact, plants have evolved various molecular mechanisms to reduce their consumption of resources and adjust their growth to adapt to adverse environmental conditions (Yamaguchi-Shinozaki and Shinozaki, 2006; Ahuja et al., 2010; Skirycz and Inze, 2010; Osakabe et al., 2011; Nishiyama et al., 2013; Ha et al., 2014).

**FIGURE 1:**
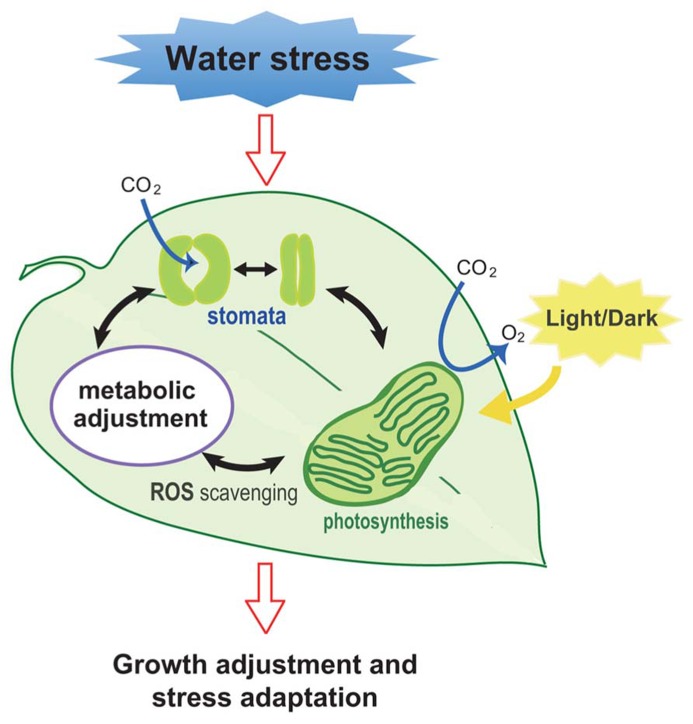
**Illustration of the response of plants to water stress.** Stomatal response, ROS scavenging, metabolic changes, and photosynthesis are all affected when plants are subjected to water stress. These collective responses lead to an adjustment in the growth rate of plants as an adaptive response for survival.

Plant growth is anchored by photosynthesis; however, excess light (EL) can cause severe damage to plants. EL induces photooxidation, which results in the increased production of highly reactive oxygen intermediates that negatively affect biological molecules and, if severe, a significant decrease in plant productivity (Li et al., 2009). Water stress that induces a decrease in leaf water potential and in stomatal opening (**Figure 1**), leading to the down-regulation of photosynthesis-related genes and reduced availability of CO_2_, has been known as one of the major factors in the EL stress (Osakabe and Osakabe, 2012).

Various molecular networks, including signal transduction, are involved in stress responses (Osakabe et al., 2011, 2013b; Nishiyama et al., 2013). The elucidation of these networks is essential to improve the stress tolerance of crops. In this review, plant responses to water stress are summarized, revealing that they are controlled by complex regulatory events mediated by abscisic acid (ABA) signaling, ion transport, and the activities of transcription factors (TFs) involved in the regulation of stomatal responses, all of which are integrated into orchestrated molecular networks, enabling plants to adapt and survive. Furthermore, recent findings on molecular mechanisms involved in protecting photosynthesis in order to adjust plant growth during water stress are discussed.

## STOMATAL SIGNALING DURING WATER STRESS

### MEMBRANE TRANSPORT AND ABA SIGNALING IN STOMATAL RESPONSES

**FIGURE 2:**
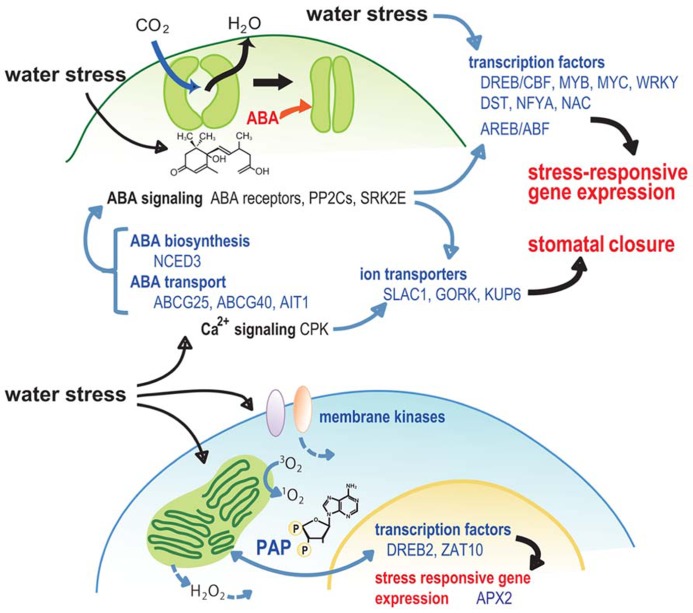
**Model for the role of signaling factors in stomatal closure and retrograde signaling during water stress**.

Stomatal activity, which is affected by environmental stresses, can influence CO_2_ absorption and thus impact photosynthesis and plant growth. In response to a water deficit stress, ion- and water-transport systems across membranes function to control turgor pressure changes in guard cells and stimulate stomatal closure. Endogenous ABA is rapidly produced during drought, triggering a cascade of physiological responses, including stomatal closure, which is regulated by a signal transduction network. 9-*cis*-epoxycarotenoid dioxygenase 3* (NCED3)* in *Arabidopsis* catalyzes a key step in ABA biosynthesis, and *NCED3* expression is rapidly induced by drought stress in a vascular tissue-specific manner (Iuchi et al., 2001; Endo et al., 2008; Behnam et al., 2013; **Figure 2**). Mutations in *nced3* reduced, while the overexpression of *NCED3* enhanced drought tolerance and/or increased WUE in several plant species (Iuchi et al., 2001; Tung et al., 2008). During drought stress, the accumulated ABA in the vascular tissue is transported to guard cells via passive diffusion in response to pH changes and by specific transporters. Two members of the membrane-localized ABC transporter family, ABCG25 and ABCG40, and one member from a nitrate transporter family, AIT1/NRT1.2/NPF4.6, have been independently isolated from *Arabidopsis* and reported as ABA transporters (Kang et al., 2010; Kuromori et al., 2010; Kanno et al., 2012; **Figure 2**). ABCG25 has a role in ABA export, whereas ABCG40 and AIT1 are involved in the import of ABA. ABA-induced stomatal closure and gene expression are reduced in the *atabcg40* mutation, resulting in reduced drought tolerance (Kang et al., 2010). These data indicate that the ABA transport system plays a significant role in water deficit tolerance and growth adjustment. Transcription of *ABCG25* was induced by ABA and drought stress, and exhibited vascular tissue-specificity (Kuromori et al., 2010). In contrast, *ABCG40* was expressed in guard cells (Kang et al., 2010), suggesting the possibility that the ABA synthesized in the vasculature during drought stress can be imported into the guard cells via these transporters. The expression pattern of *AIT1/NRT1.2/NPF4.6* was similar to *ABCG25 *and also showed vascular tissue-specificity (Kanno et al., 2012). This finding suggests that ABA import systems in vascular tissues may also play an important role in the regulation of water stress responses.

In response to drought stress, ABA stimulates a signaling pathway that triggers the production of reactive oxygen species (ROS), which in turn induces an increase in cytosolic Ca^2^^+^. Subsequently, two distinct types of anion channels, a slow-activating sustained (S-type), and a rapid-transient (R-type), are activated and the anion efflux results in a depolarization of the plasma membrane. This leads to a decrease in the inward K^+^ channels (KAT1/KAT2) and H^+^-ATPase, which are involved in stomatal opening, and the activation of outward K^+^ channels, including GUARD CELL OUTWARD RECTIFYING K^+^ CHANNEL (GORK) that has a role in K^+^ efflux. The anion and K^+^ efflux from guard cells results in a reduction of guard cell turgor which causes stomatal closure (Schroeder and Hagiwara, 1989; Pei et al., 1997; Kwak et al., 2003; Negi et al., 2008; Vahisalu et al., 2008). *SLAC1* (*SLOW ANION CHANNEL-ASSOCIATED 1*) functions as a major S-type anion channel in guard cells (Negi et al., 2008; Vahisalu et al., 2008), and is activated directly by a Snf1-related protein kinase 2 (SRK2E/OST1/SnRK2.6). This kinase is involved in the ABA-signaling complex of the ABA receptor, PYR family and PP2Cs (Geiger et al., 2009; Lee et al., 2009). S-type anion channels are also activated by the calcium-dependent protein kinases CPK3, CPK6, CPK21, and CPK23 (Geiger et al., 2010; Brandt et al., 2012). KAT1 has also been shown to be a direct target of regulation by ABA, since its activity is directly inhibited via phosphorylation by an ABA-activated SRK2E (Sato et al., 2009). Recently, the activity of KUP6, a KUP/HAK/KT family K^+^ transporter, has also been shown to be involved in the direct regulation during drought stress via phosphorylation by an ABA-activated SRK2E (Osakabe et al., 2013a). These results suggest that the complicated, but direct, control of ion transport systems by ABA may play an important role in stomatal responses that impact the tolerance of plants to water stress and influence plant growth (**Figure 2**).

### TRANSCRIPTION FACTORS

The expression of various genes with functions in the water deficit responses, are specifically induced during the stress. Transcriptomic and proteomic analyses in various species have identified the involvement of general physiological processes associated with drought-responsive gene expression (Molina et al., 2008; Aprile et al., 2009; Walia et al., 2009; Abebe et al., 2010; Dugas et al., 2011; Jogaiah et al., 2012; Le et al., 2012; Utsumi et al., 2012). These studies have identified the conserved, as well as, species-specific regulatory and functional drought-responsive genes, including osmoprotectants and ABA biosynthesis, late embryogenesis abundant (LEA) and chaperone, ROS-related, ion homeostasis, and signaling genes. Additionally, key TFs regulating drought-responsive gene transcription have also been identified, such as MYB, MYC, DREB/CBF (drought-responsive *cis*-element binding protein/C-repeat-binding factor), ABF/AREB, NAC, and WRKY TFs (Stockinger et al., 1997; Sakuma et al., 2006; Tran et al., 2007b; Nakashima et al., 2009; Ishida et al., 2012; **Figure 2**). Corresponding *cis*-motifs, DRE/CRT and ABRE (ABA-responsive *cis*-element), have also been discovered in the promoters of many stress-responsive genes (Yamaguchi-Shinozaki and Shinozaki, 2006).

ABA-responsive *cis*-element-mediated transcription via ABF/AREB is directly regulated by an ABA receptor complex involving SnRK2 that activate ABF/AREBs by phosphorylation (Umezawa et al., 2010). The action of SnRK2 represents one of the important mechanisms regulating the rapid, adaptive response of plants to drought. DREB and AREB activate the transcription of various genes that are expressed in variety tissues. Additionally, novel types of TFs, with critical functions in stomatal responses, have also been identified. DST (drought and salt tolerance), a C_2_H_2_-type TF, controls the expression of genes involved in H_2_O_2_ homeostasis, and mediates ROS-induced stomatal closure and abiotic stress tolerance in rice (Huang et al., 2009). Drought-inducible nuclear TF, NFYA5, was reported to control stomatal aperture and play a role in drought tolerance in *Arabidopsis* (Li et al., 2008). *SNAC1* (*STRESS-RESPONSIVE NAC1*) is expressed in rice guard cells, and overexpression of this gene enhanced ABA sensitivity, stomatal closure, and both DST in rice (Hu et al., 2006). *AtMYB60 *and* AtMYB61* are expressed mainly in guard cells, and important TFs regulating stomatal aperture and drought tolerance in plants (Cominelli et al., 2005). AtMYB60 is a negative regulator of stomatal closure (Cominelli et al., 2005; Liang et al., 2005). Further studies to determine the molecular targets and signaling systems associated with these TFs in stomatal responses will increase our understanding of the regulatory networks controlling plant drought responses and growth adjustment.

## EARLY WATER STRESS RESPONSE AND SIGNAL TRANSDUCTION PATHWAYS

Receptor and sensor proteins localized to membranes play important roles in various signaling pathways, conveying information to their cytoplasmic target proteins via catalytic processes, such as phosphorylation. Plasma membrane signaling has been hypothesized to be involved in the initial process of water status perception outside the cell (Maathuis, 2013). AHK1, an *Arabidopsis* histidine kinase (HK) localized to the plasma membrane mediates osmotic-stress signaling in prokaryotes and has been shown to function as an osmosensor. Overexpression of *AHK1* enhanced drought tolerance in *Arabidopsis* (Urao et al., 1999; Tran et al., 2007a). *ahk1* mutants exhibited decreased sensitivity to ABA and the downregulation of ABA- and/or stress-responsive genes, indicating that AHK1 acts as an osmosensor and functions as a positive regulator of osmotic-stress signaling (Tran et al., 2007a; Wohlbach et al., 2008). Downstream AHK1 cascades appear to be controlled by AHPs and ARRs as part of a multiple His-Asp phosphorelay. However, the factors that receive signals from AHK1, and also the precise composition of the signaling cascades, remain to be determined. In contrast, in *Arabidopsis*, the cytokinin (CK) receptor HKs, AHK2, AHK3, and AHK4, have been shown to negatively regulate ABA and drought signaling (Tran et al., 2007a, 2010). Multiple mutants of *ahk2*, *ahk3*, and *ahk4 *display increased sensitivity to ABA and enhanced tolerance to drought (Tran et al., 2007a; Jeon et al., 2010). These findings indicate the existence of crosstalk among ABA, CK, and stress-signaling pathways****(Nishiyama et al., 2011; Ha et al., 2012).

In *Arabidopsis*, the receptor-like kinase (RLK) family includes more than 600 members, with the leucine rich-repeat (LRR)-RLKs constituting the largest subgroup (Gish and Clark, 2011). Several RLKs localized to the plasma membrane are known to be involved in the early steps of osmotic-stress signaling in a variety of plant species (Osakabe et al., 2013b). These stress-related RLKs possess a number of different extracellular domains (e.g., LRR, an extensin-like domain, or a cysteine-rich domain; Bai et al., 2009; de Lorenzo et al., 2009; Osakabe et al., 2010a; Yang et al., 2010; Tanaka et al., 2012), indicating that different environmental stimuli may activate RLK-mediated signaling pathways and convey the osmotic conditions outside of the cells. RLKs that bind to cell-walls, such as cell wall-associated kinases (WAKs), the proline-rich extensin-like receptor kinase (PERKs; Osakabe et al., 2013b), and the CrRLKs (*Catharanthus roseus* RLK1-like family; Schulze-Muth et al., 1996) have recently been predicted to be involved in the perception of turgor pressure (Steinwand and Kieber, 2010; Christmann et al., 2013). A potential link between the RLKs in cell-wall binding, ABA biosynthesis and water stress response could be determined by analyzing their roles in signaling systems associated with specific mechanosensing pathways activated in response to water stress. This would shed light on the early signaling system controlling water stress tolerance and growth adjustment.

## PROTECTING PHOTOSYNTHESIS DURING WATER STRESS

Water stress directly affects rates of photosynthesis due to the decreased CO_2_ availability resulted from stomatal closure (Flexas et al., 2006; Chaves et al., 2009), and/or from changes in photosynthetic metabolism (Lawlor, 2002). EL has a negative effect on photosynthesis when the rates of photosynthesis are reduced by water stress (Li et al., 2009; Osakabe and Osakabe, 2012). A strong interconnection between the responses to EL and drought stresses has been suggested, and around 70% genes induced by EL are also induced by drought (Kimura et al., 2002; Chan et al., 2010; Estavillo et al., 2011). EL also stimulates the production of ROS, such as H_2_O_2_, superoxide (O_2_^-^) and singlet oxygen (^1^O_2_), by specific photochemical and biochemical processes, which also exerts deleterious effects on photosynthesis (Li et al., 2009). H_2_O_2_ induces the up-regulation of a variety of genes that overlap with genes up-regulated by various chemical and environmental stresses, such as methyl viologen, heat, cold, and drought (Vandenabeele et al., 2004; Vanderauwera et al., 2005). The transcription of cytosolic ascorbate peroxidase encoding genes (*APXs*), which have important roles in the scavenging of cytosolic H_2_O_2_, responds positively to EL stress and the redox state of plastoquinone (PQ; Karpinski et al., 1997). *APX* loss-of-function mutants exhibited an accumulation of degraded chloroplast proteins, indicating that APXs play a protective role as ROS scavengers for chloroplast proteins under EL conditions (Davletova et al., 2005; Li et al., 2009). *AtAPX2 *was also induced by drought stress and ABA (Rossel et al., 2006), suggesting that APX mediates ROS scavenging in response to both EL and water stress. A gain-of-function mutant, *altered apx2 expression 8* (*alx8*), which has constitutively higher levels of *APX2* expression, exhibited improved WUE and drought tolerance (Rossel et al., 2006; Wilson et al., 2009; Estavillo et al., 2011). In *Arabidopsis*, the zinc-finger TFs, *ZAT10* and *ZAT12*, are induced in plants acclimated to EL or ROS treatment. The overexpression of *ZAT10* and *ZAT12* highly induced expression of various stress-related genes, including *APX*s (Rizhsky et al., 2004; Davletova et al., 2005; Rossel et al., 2007). Several transgenic lines that overexpressed *ZAT10* exhibited enhanced drought stress tolerance (Sakamoto et al., 2004). ZAT10 and ZAT12 regulate the responses to EL and drought stresses, which are mediated by ROS (Davletova et al., 2005; Mittler et al., 2006), suggesting their potential roles in protecting photosynthesis from the injury during water stress (**Figure 2**).

Plants can monitor chloroplast status by plastid-to-nucleus signals, as plastid-to-nucleus retrograde signaling. This signaling system can regulate the expression of genes that function in the chloroplast. The retrograde signaling plays an important role in regulating the chloroplastic processes and also in the adaptive responses to environmental stresses (Chan et al., 2010). Chlorophyll intermediates, such as Mg-protoporphyrin IX (Mg-Proto), control the expression of nuclear genes in plants exposed to EL conditions, acting as a retrograde signal. The *genomes uncoupled* (*gun*) mutants, *gun4* and *gun5*, exhibit impaired generation of Mg-Proto that has been shown to act as a signal to repress *LHCB* gene expression in *Arabidopsis* (Mochizuki et al., 2001; Strand et al., 2003; Pontier et al., 2007). *LHCB* expression is also controlled by GUN1 and ABI4 (ABSCISIC ACID-INSENSITIVE 4) that encodes a TF involved in ABA signaling (Koussevitzky et al., 2007). Collectively, these factors are thought to be involved in multiple retrograde signaling pathways. Moulin et al. (2008) re-examined the proposed role of Mg-Proto and other chlorophyll intermediates as signaling molecules and reported that none of the intermediates could be detected in ROS-induced plants under conditions where nuclear gene expression was repressed. The authors hypothesized that Mg-Proto (which accumulates in a light-dependent manner) is extremely short-lived and may generate ^1^O_2_ under EL conditions; however, a much more complex ROS signal may be generated during chloroplast degradation. There is increasing evidence for the regulation of nuclear gene expression by ^1^O_2_ (op den Camp et al., 2003) and H_2_O_2_ (Kimura et al., 2003). However, a clear role for these ROS molecules, either individually or in combination, requires further investigation.

Recently, several novel retrograde signaling pathways have been identified, including the 3′-phosphoadenosine 5′-phosphate (PAP) pathway, which is regulated by SAL1/ALX8/FRY1, and the methylerythritol cyclodiphosphate (MEcPP) pathway (Estavillo et al., 2011; Xiao et al., 2012). PAP has been described as a chloroplast to nuclear mobile signal that regulates gene expression. *ALX8* encodes a phosphatase that converts PAP to AMP and regulates PAP levels (Wilson et al., 2009; Estavillo et al., 2011). *alx8* mutant exhibited drought-tolerant phenotypes and constitutive upregulation of approximately 25% of the EL-regulated transcriptome, suggesting that SAL1/ALX8/FRY1 can act as a component of both EL and drought signaling networks, and that the SAL1-PAP retrograde pathway can alter nuclear gene expression during EL and drought (Rossel et al., 2006; Wilson et al., 2009; Estavillo et al., 2011; **Figure 2**). MEcPP is a precursor of isoprenoids generated by the methylerythritol phosphate (MEP) pathway, and can induce expression of nuclear encoded stress-responsive genes (Xiao et al., 2012). MEcPP is induced by various abiotic stresses, such as high light and wounding, and has been proposed to act as a retrograde signal in response to these stresses (Xiao et al., 2012). Evidence from the above studies suggests that metabolite signals, whose levels are influenced by environmental conditions, are used to establish an interaction between plastids and the nucleus and regulate chloroplast function to adjust plant growth in response to various stresses, including drought.

## CONCLUSION AND FUTURE PERSPECTIVE

Due to the sessile life cycle, plants have evolved mechanisms to respond and adapt to adverse environmental stresses during their development and growth. Plant growth is impaired by severe drought stress due to a decrease in stomatal opening, which limits CO_2_ uptake and hence reduces photosynthetic activity. In order to develop strategies to maintain plant productivity, it is essential to understand the various regulatory mechanisms that control and enhance adaptive responses to stress in different plant species. In this review, we focused on the molecular mechanisms involved in the plant responses to water stress and the concomitant growth adjustment. These mechanisms include stomatal responses, ion transport, activation of stress signaling pathways, and responses to protect photosynthesis from injury. Understanding these key factors will enable us to improve plant productivity during water stress.

In parallel with the identification of the key molecular factors involved in these mechanisms, new technologies to bioengineer superior plants will also enable the development of plants with improved plant productivity. Although transgenic approaches have been effectively used to develop plant genotypes with improved stress tolerance under field conditions, estimation of the desired effects and their stability over many generations is required. Mutagenesis has also been used in plant breeding for a long time to create genetic variation; however, it takes considerable resources and effort to generate genotypes with the desired phenotype due to the random nature of the introduction of mutations. Recently, genome editing technology has made remarkable advances in the ability to modify the genome in a site-specific manner. Genome editing technology utilizes custom-designed restriction endonucleases, such as zinc finger nucleases (ZFN) or TAL-effector nucleases (TALEN; Shukla et al., 2009; Osakabe et al., 2010b; Zhang et al., 2010; Cermak et al., 2011), and more recently, the CRISPR/CAS system (Li et al., 2013; Nekrasov et al., 2013; Shan et al., 2013). Utilization of this technology will make it possible to modify the regulation of key genes that will convey improved stress tolerance while maintaining productivity. Further studies using new molecular approaches, including the identification of gene variants associated with the significant agronomic traits, will facilitate the molecular engineering of plants with increased tolerance to severe environmental stresses.

## Conflict of Interest Statement

The authors declare that the research was conducted in the absence of any commercial or financial relationships that could be construed as a potential conflict of interest.
